# A Rare Case of Spontaneous Recurrent Cerebrospinal Fluid Rhinorrhea

**DOI:** 10.7759/cureus.3883

**Published:** 2019-01-14

**Authors:** Wen Wang, Emily Emmet, Alexander Leyva, John V Dennison, Sean Dodson

**Affiliations:** 1 Internal Medicine, Florida Hospital, Orlando, USA; 2 Family Medicine, Florida Hospital, Orlando, USA; 3 Radiology, Florida Hospital, Orlando, USA

**Keywords:** ct cisternography, endonasal endoscopic repair, high pressure csf leaks, meningitis, multiple defect csf leak, recurrent cerebrospinal fluid rhinorrhoea, β-2-transferrin

## Abstract

Cerebrospinal fluid (CSF) rhinorrhea is most commonly associated with preceding trauma. Spontaneous CSF rhinorrhea has rarely been documented. Clinical, biochemical, and radiological examination are necessary to establish its diagnosis. Detection of beta-2 transferrin in watery nasal discharge is diagnostic for the presence of CSF. Computed tomography (CT) cisternography or magnetic resonance imaging (MRI) cisternogram are confirmatory radiologic modalities for localization of the leakage site.

## Introduction

The most frequent cause of cerebrospinal fluid (CSF) rhinorrhea is iatrogenic or traumatic. Spontaneous CSF rhinorrhea has rarely been reported [1]. Here we discuss a patient case that focuses on the presentation, diagnosis, and treatment of spontaneous CSF rhinorrhea.

## Case presentation

A 52-year-old otherwise healthy female presented with clear nasal discharge. The nasal discharge reportedly began happening in 2016 and spontaneously resolved. It resumed in April 2018 with no inciting events. The watery nasal discharge worsened with bending over, activity, and coughing. She reported that the nasal discharge was associated with headache as well as a continuous salty taste in her mouth and a feeling of fullness in her ear. The patient denied any trauma. A review of systems was negative except for the headaches and nasal discharge. The patient denied a history of any connective tissue disease, rhinitis, or recurrent sneezing. On physical exam no other abnormalities were noted other than a clear liquid coming from the right side of her nose. Her neurological examination was unremarkable as well.

**Figure 1:**
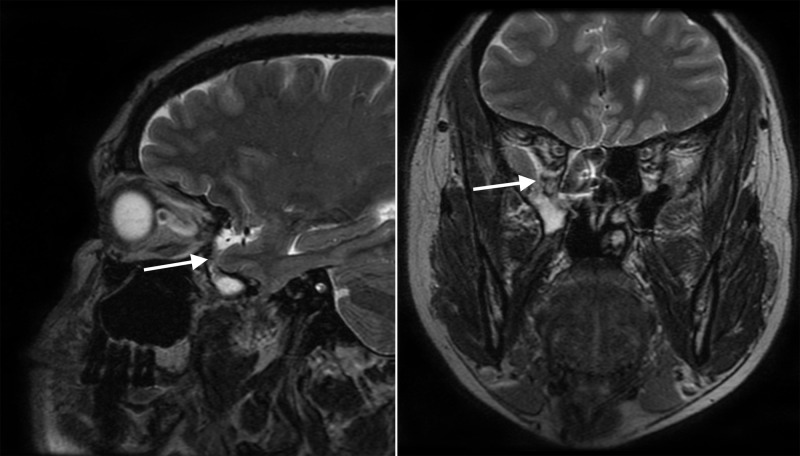
MRI of the brain without contrast Magnetic resonance imaging (MRI) of the brain demonstrates an empty sella, intracranial hypertension, and a right middle fossa encephalocele associated with a possible CSF fistula.

**Figure 2:**
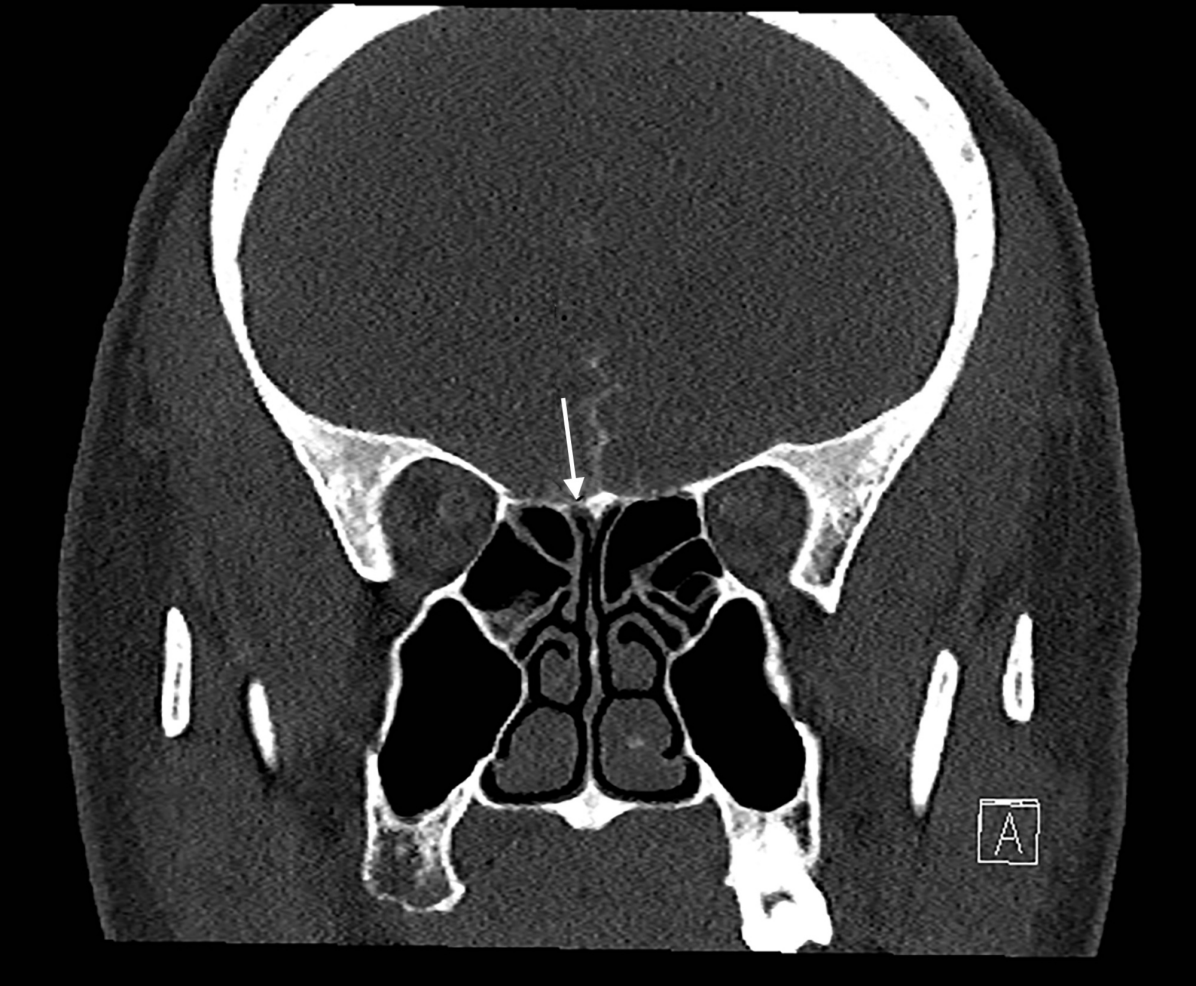
CT of the cribriform plate, coronal view Computed tomography (CT) reveals a severe thinning of the cribriform plate and fovea ethmoidalis bilaterally, the right worse than the left with multiple suspected small focal regions of cortical discontinuity along the right cribriform plate and fovea ethmoidalis.

**Figure 3:**
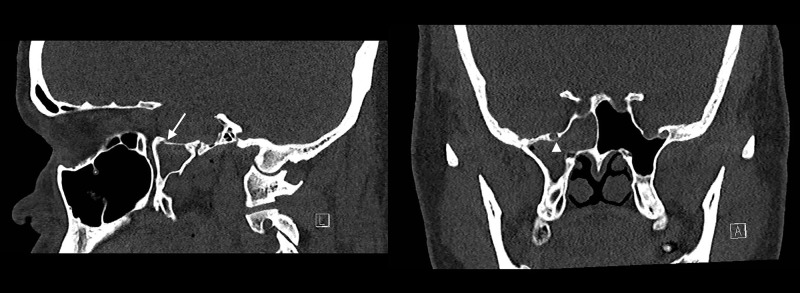
CT pre-contrast panel Computed tomography (CT) shows a discrete focal region of severe bone thinning and probable cortical discontinuity within the anterior, inferior aspect of the right middle cranial fossa just lateral to the foramen rotundum. The contrast material within the middle cranial fossa appears to extend through this defect into the lateral aspect of the right sphenoid sinus.

**Figure 4:**
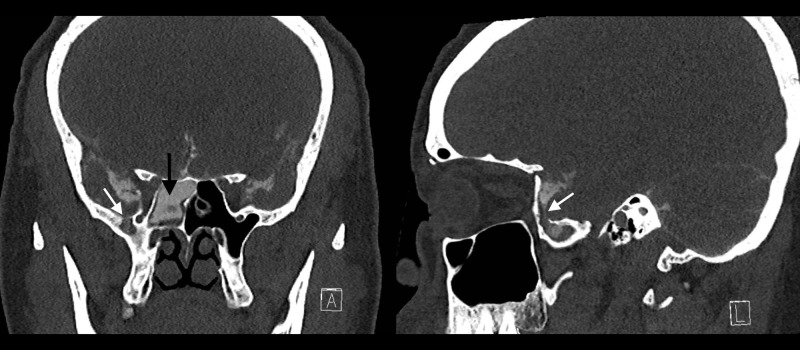
CT cisternogram post-contrast, coronal and sagital view Computed tomography (CT) shows that the contrast material within the middle cranial fossa appears to extend through this defect into the lateral aspect of the right sphenoid sinus.

Testing of the fluid for beta-2 transferrin was positive, indicating that the fluid was CSF. An MRI of the brain showed an empty sella, intracranial hypertension, and a right middle fossa encephalocele associated with a possible CSF fistula (Figure 1). A CT cisternogram was performed to confirm the diagnosis, which revealed a discrete focal region of severe bone thinning and probable cortical discontinuity in multiple locations within the anterior, inferior aspect of the right middle cranial fossa just lateral to the foramen rotundum, cribriform plate, and bilateral fovea ethmoidalis (Figures 2-4). The Neurosurgery and Ear Nose Throat (ENT) departments were consulted. Endonasal repair was recommended and the patient was instructed to follow up in the clinic for elective surgery.

## Discussion

Most CSF rhinorrhea cases have traumatic or iatrogenic etiologies. However, our case report is a rare presentation of spontaneous CSF rhinorrhea [1]. The etiology for this may be attributed to increased intracranial pressure (ICP), which is supported by imaging findings of coexistent pseudotumor cerebri. It is also associated with the presence of meningoceles or encephaloceles. The risk factors include middle age, obesity, and hypertension [2]. Detection of beta-2 transferrin in the watery nasal discharge is diagnostic of CSF [3]. Several radiological modalities are available to identify anatomical CSF rhinorrhea sites. HRCT (high-resolution computed tomography) can provide better anatomical details than routine CT and is a reasonable choice as an initial test. Cisternograms, which include CT cisternogram, MRI cisternogram, and radionuclide cisternogram, are tests of choice to confirm CSF leak [4]. There is evidence suggesting that DECT (dual energy CT scan) combined with CT cisternogram provides higher sensitivity and specificity compared to other modalities [5]. To prevent meningitis from the defect, surgical repair is recommended. The preferred approach is through endonasal endoscopy, the benefits of which include minimal morbidity, short hospital stay, and preservation of olfaction [6,7].

## Conclusions

Spontaneous CSF leak is a rare cause of CSF rhinorrhea. The diagnosis of CSF rhinorrhea is established with the biochemical testing of the fluid for beta-2 transferrin and imaging tests using CT cisternogram. Surgical repair, preferably using an endonasal approach, is indicated to prevent the complication of meningitis.
